# Mild angle early onset idiopathic scoliosis avoid progression under fits physiotherapy

**DOI:** 10.1186/1748-7161-9-S1-O29

**Published:** 2014-12-04

**Authors:** Marianna Białek

**Affiliations:** 1 FITS Center, Jawor, Poland

## Background

Physiotherapy for stabilization of idiopathic scoliosis angle in growing children remains controversial. Specially, little data on effectiveness of physiotherapy in children with Early Onset Idiopathic Scoliosis (EOIS) was published.

## Aim

The aim was to check results of FITS physiotherapy in a group of children with EOIS.

## Methods

The charts of the patients archived in a prospectively collected database were retrospectively reviewed. The inclusion criteria were: diagnosis of Early Onset Idiopathic Scoliosis based on spine radiography, age below 10 years, both girls and boys, Cobb angle between 11° and 30°, Risser zero, FITS therapy, no other treatment (nighttime bracing etc.), follow-up minimum 2 years from the initiation of the treatment. The criterion for curve progression was Cobb angle increase of 6° or more at any follow-up radiograph. The criterion for curve stabilization was the Cobb angle within the range ± 5° comparing to the initial radiograph. The criterion for curve correction was Cobb angle decrease of 6° or more at the final follow-up radiograph.

## Results

There were 41 children with Early Onset Idiopathic Scoliosis, 36 girls and 5 boys, mean age 7.7 ±1.3 years (range 4 to 9 years) who started FITS therapy. The curve pattern was single thoracic (5 children), single thoracolumbar (22 children) or double thoracic/thoracolumbar (14 children), totally 55 structural curvatures. The minimum follow-up was 2 years after initiation of the FITS treatment, maximum was 16 years, mean 4.8 years). At follow-up the mean age was 12.5 ±3.4 years. Out of 41 children, 10 passed pubertal growth spurt at the final follow-up while 31 were still immature and continued FITS therapy. Out of 41 children, 27 improved, 13 were stable and one progressed. Out of 55 structural curves, 32 improved, 22 were stable and one progressed. For the 55 structural curves, the Cobb angle significantly decreased from 18.0° ± 5.4° at first assessment to 12.5° ± 6.3° at last evaluation, p<0.0001, paired t-test. The Angle of Trunk Rotation decreased significantly from 4.7° ± 2.9° to 3.2° ± 2.5° at last evaluation, p<0.0001, paired t-test.

## Conclusion

FITS physiotherapy was effective in preventing curve progression in children with Early Onset Idiopathic Scoliosis. Final post-pubertal follow-up data are needed.

